# Comprehensive Behavioral Analysis of Male *Ox1r*^−/−^ Mice Showed Implication of Orexin Receptor-1 in Mood, Anxiety, and Social Behavior

**DOI:** 10.3389/fnbeh.2015.00324

**Published:** 2015-12-10

**Authors:** Md. G. Abbas, Hirotaka Shoji, Shingo Soya, Mari Hondo, Tsuyoshi Miyakawa, Takeshi Sakurai

**Affiliations:** ^1^Department of Molecular Neuroscience and Integrative Physiology, Faculty of Medicine, Kanazawa UniversityKanazawa, Japan; ^2^Division of Systems Medical Science, Institute for Comprehensive Medical Science, Fujita Health UniversityToyoake, Japan; ^3^International Institute for Integrative Sleep Medicine, University of TsukubaTsukuba, Japan; ^4^Section of Behavior Patterns, Center for Genetic Analysis of Behavior, National Institute for Physiological SciencesOkazaki, Japan

**Keywords:** orexin, orexin receptor 1 (OX1R), emotion, anxiety, social behavior, hypocretin

## Abstract

Neuropeptides orexin A and orexin B, which are exclusively produced by neurons in the lateral hypothalamic area, play an important role in the regulation of a wide range of behaviors and homeostatic processes, including regulation of sleep/wakefulness states and energy homeostasis. The orexin system has close anatomical and functional relationships with systems that regulate the autonomic nervous system, emotion, mood, the reward system, and sleep/wakefulness states. Recent pharmacological studies using selective antagonists have suggested that orexin receptor-1 (OX1R) is involved in physiological processes that regulate emotion, the reward system, and autonomic nervous system. Here, we examined *Ox1r*^−/−^ mice with a comprehensive behavioral test battery to screen additional OX1R functions. *Ox1r*^−/−^ mice showed increased anxiety-like behavior, altered depression-like behavior, slightly decreased spontaneous locomotor activity, reduced social interaction, increased startle response, and decreased prepulse inhibition. These results suggest that OX1R plays roles in social behavior and sensory motor gating in addition to roles in mood and anxiety.

## Introduction

Hypothalamic neuropeptides orexins, orexin A and orexin B, which are also known as hypocretin 1 and hypocretin 2, respectively (de Lecea et al., 1998; Sakurai et al., 1998), have been shown to be important factors for maintaining sleep/wakefulness and regulating feeding behavior, emotion, the reward system, and energy homeostasis (Yamanaka et al., 2003; Harris et al., 2005; Sakurai et al., 2005; Sakurai, 2007, 2014; Tsujino and Sakurai, 2009; Sakurai and Mieda, 2011). Orexin-producing neurons (orexin neurons) send projections widely throughout the central nervous system (CNS), including the cerebral cortex, limbic system [including the amygdala, bed nucleus of the stria terminalis (BST) and hippocampus], hypothalamus [such as the arcuate nucleus (ARC) and tuberomammillary nucleus (TMN)], and brain stem areas including the central gray, locus coeruleus (LC), and dorsal raphe (DR) (Peyron et al., 1998; Date et al., 1999; Nambu et al., 1999).

There are two orexin receptor subtypes, orexin receptor 1 (OX1R) and orexin receptor 2 (OX2R). OX1R exhibits higher affinity to orexin A over orexin B, while OX2R shows similar affinities to both isopeptides (Sakurai et al., 1998). Expression of OX1R is observed in various areas of the brain including the prefrontal and infralimbic cortex, hippocampus, amygdala, BST, paraventricular thalamic nucleus (PVT), anterior hypothalamus, DR, ventral tegmental area (VTA), LC, and laterodorsal tegmental nucleus (LDT)/pedunculopontine nucleus (PPT) (Trivedi et al., 1998; Lu et al., 2000; Marcus et al., 2001; Tsujino and Sakurai, 2009; Mieda et al., 2011). Expression of OX2R is observed in the amygdala, BST, PVT, DR, VTA, and LDT/PPT (Lu et al., 2000; Marcus et al., 2001; Mieda et al., 2011). Many studies using *OX2R*^−/−^ mice have suggested important physiological roles of OX2R in the maintenance of wakefulness states (Hondo et al., 2010; Sakurai and Mieda, 2011) and the regulation of metabolic states and feeding behavior (Funato et al., 2009).

Recently, studies using selective OX1R antagonists have suggested that OX1R plays important roles in the regulation of feeding behavior, the reward system, emotion, and the autonomic nervous system (Sakurai, 2014). On the other hand, only limited information is available thus far regarding the phenotype of OX1R-deficient mice, although a recent study suggested a role of this subtype in formation of emotional memory and emergence of fear-related behaviors (Soya et al., 2013). In order to obtain further information about the physiological roles of OX1R, we performed a series of behavioral tests on mice lacking the orexin-1 receptor (*Ox1r*^−/−^ mice). We found that *Ox1r*^−/−^ mice showed altered depression-like behavior, increased anxiety-like behavior, impairment of sensorimotor gating, abnormal social behavior, and decreased locomotor activity compared with the wild-type control mice. Collectively, this study suggests that OX1R might be involved in regulation of mood and anxiety.

## Materials and methods

### Animals

All experimental procedures used in this study were approved by the Animal Experiment and Use Committee of Kanazawa University (AP-111947), and were in accordance with National Institute of Health (NIH) guidelines. *Ox1r*^−/−^ mice (Soya et al., 2013) were obtained by mating of heterozygous *Ox1r*^+/−^ mice. Genotyping of these mice was performed by PCR using DNA samples prepared from the tails. We used the following primers for genotyping; common, 5′-CTCTTTCTCCACAGAGCCCAGGACTC-3′, wild-type, 5′-GCAAGAATGGGTATGAAG GGAAGGGC-3′, and knockout, 5′-TGAGCGAGTAACAACCCGTCGGATTC-3′. The mice were backcrossed to wild-type C57Bl/6J mice at least 10 times. The wild-type littermates of the mutants were used as controls. To minimize a “litter effect,” two to five pups from four litters were used in the experiments. *Ox1R*^−/−^ and wild-type mice were group-housed four per cage (one to two *Ox1R*^−/−^s and two to three wild-types in a cage). To generate the *Ox1R*^−/−^ and wild-type mice, we mated four dams with one male per cage at the time of mating. Pups from the four dams were group-housed in a cage until weaning. Only two to five pups per four dams (from a mating cage) were transferred to a cage at the time of weaning and used in this study. The mice were transferred to Fujita Health University from Kanazawa University at the age of 8–10 weeks. Mice were maintained under a strict 12 h light/dark cycle (lights on at 7:00 at Fujita Health University) in a temperature- and humidity-controlled room. Food and water were available *ad libitum*. Two weeks after arrival, mice were subjected to a battery of behavioral tests. All behavioral testing procedures were approved by the Institutional Animal Care and Use Committee of Fujita Health University. All efforts were made to minimize the animals' suffering and discomfort and to reduce the number of animals used.

### Behavioral experiments

All behavioral experiments were performed during the light phase (9:00–16:00). Only male mice were used. Mice were group-housed, four mice per cage. Behavioral experiments of this study were performed as previously described (Miyakawa et al., 2003). General health screening including measurement of body weight and body temperature were also conducted (Miyakawa et al., 2001b, 2003).

### Neuromuscular strength test

Neuromuscular strength of the mice was examined in the grip strength and wire hang tests. In order to assess forelimb grip strength, we used a grip strength meter (O'Hara & Co., Tokyo, Japan). In this test, we lifted the mice and held them by their tail. As a result mice could grasp a wire grid with their forepaws. Next, we gently pulled the mice backward with their tail after maintaining a posture parallel to the table surface until they released the grid. Each mouse was tested three times and the highest value of grip strength (Newton) was used for analysis. In the wire hang test, after placing mice on a wire mesh, it was gently inverted and waved about. Mice gripped the wire to stop themselves falling off. Latency to fall off the wire mesh was recorded (Tsujimura et al., 2008).

### Rotarod test

The test was performed to examine motor coordination and balance (Miyakawa et al., 2003). This was performed using an accelerating rotarod (UGO Basile Accelerating Rotarod) where a mouse was placed on the rotating drum (3 cm diameter). The time each animal was able to maintain its balance on the rotating drum was measured. The starting speed of the rotarod was 4 rpm, maximum was 40 rpm, and the test duration was 5 min.

### Hot plate test

We performed a hot plate test to evaluate sensitivity to a painful stimulus or nociception (Miyakawa et al., 2003). Mice were placed on a hot plate (Columbus Instruments, Columbus, OH) with a temperature of 55.0 ± 0.3°C, and latency to the first paw response (sec) was recorded manually (cut-off time: 15 s). The paw response was either a foot shake or a paw lick.

### Light/dark transition test

We used an apparatus consisting of a cage (21 × 42 × 25 cm) which was divided into two equal compartments by a black partition containing a small door (O'Hara & Co., Tokyo) (Takao and Miyakawa, 2006). One compartment was brightly illuminated (390 lux) and the other was dark (2 lux). Mice were placed into the dark side and allowed to move freely between the two chambers through the small door for 10 min. The distance traveled, total number of transitions, time spent in the light chamber, and latency to enter the light chamber were recorded automatically using ImageLD software.

### Elevated plus maze test

We used an apparatus consisting of two open arms and two closed arms. The open arms (25 × 5 cm) were surrounded by 3-mm high Plexiglas ledges to minimize the likelihood of mice falling down from the apparatus. The closed arms were the same size, with 15 cm high transparent walls (O'Hara & Co, Tokyo) (Miyakawa et al., 2003). This apparatus was made of white plastic. It was elevated 55 cm above the floor. Each mouse was placed in the central area of the maze (5 × 5 cm), facing one of the closed arms. Mouse behavior was recorded during a 10-min test period. The number of entries into arms, time spent in open arms, and distance traveled (cm) were recorded. Data were analyzed automatically using ImageEP software.

### Social interaction test in a novel environment

The social interaction test in a novel environment was performed in two mice of the same genotype, which were previously kept in different cages. They were placed into a box (40 × 40 × 30 cm) together and allowed to move freely for 10 min (Miyakawa et al., 2003). Mouse behavior was analyzed automatically using ImageSI software. The automatic scoring by the ImageSI was validated by comparing it with manual scoring by actual observation of a well-trained experimenter (weight-matched male C57BL/6J pairs, *n* = 8: for the total duration of contacts, *r* = 0.978, *p* < 0.0001; for the number of contacts, *r* = 0.953, *p* < 0.0001). The total duration of contacts (sec), number of contacts, total duration of active contacts (sec), mean duration per contact (sec), and distance traveled (cm) were measured.

### Porsolt forced swim test

We used an apparatus consisting of transparent plastic cylinders (20 cm height × 10 cm diameter) (Miyakawa et al., 2003). The cylinders were filled with water (22–23°C) up to a level of 7.5 cm. Mice were put into the cylinders, and their immobility behavior and distance traveled (cm) were recorded over a 10-min test period on Day 1 and Day 2. Data acquisition and analysis were performed automatically using ImagePS software.

### Startle response/prepulse inhibition test

A startle reflex measurement system was used (O'Hara & Co, Tokyo) for assessing acoustic startle response to loud noises and the prepulse inhibition of the acoustic startle response. The test was conducted as previously described (Miyakawa et al., 2003; Takao et al., 2013; Nakao et al., 2015).

### Sociability and social novelty preference test

Sociability and social novelty preference test is performed according to a slightly modified protocol of Moy et al. (2004). The apparatus consisted of a rectangular, three chambered box and a lid with an infrared video camera (O'Hara & Co., Tokyo). Each chamber was 20 × 40 × 47 cm and the dividing walls were made from clear Plexiglas with small square openings (5 × 3 cm) allowing access into each chamber. Each mouse were placed in the box for 10 min and allowed freely explored to habituate it. Second, in the sociability test, an unfamiliar C57BL/6J male mouse (stranger 1), that had no prior contact with the subject mice, was put into one of the wire cages (9 cm in diameter, 11 cm in height, vertical bars 0.5 cm apart) that were located in the corners of each lateral compartment. The stranger mouse was enclosed in a small round wire cage, which allowed nose contact between the bars, but prevented fighting. The subject mouse was placed in the middle chamber and allowed to explore the entire box for a 10-min session (sociability test). After the 10-min test session, a second unfamiliar mouse (stranger 2) was placed in the previously empty but otherwise identical small wire cage in the opposite chamber. The test mouse thus had a choice between the first, already-investigated unfamiliar mouse, and the novel unfamiliar mouse (social novelty preference test). The amount of time spent in each chamber and of time spent around each cage during the first and second 10-min sessions were measured. Data acquisition and analysis were performed automatically using ImageCSI software.

### Open field test

Open field test was performed to measure locomotor activity (Miyakawa et al., 2001a, 2003; Takao et al., 2008; Tsujimura et al., 2008). Each mouse was placed in the corner of an open field apparatus (40 × 40 × 30 cm; Accuscan Instruments, Columbus, OH, USA). The distance traveled (cm), vertical activity, time spent in the center (sec), and beam–break counts for stereotypic behaviors were recorded and analyzed accordingly.

### Tail suspension test

The tail suspension test was performed for a 10-min test session according to the procedures described previously (Steru et al., 1985). Mice were suspended from 30 cm above the floor in a visually isolated area by adhesive tape placed approximately 1 cm from the tip of the tail. Their behavior was recorded and analyzed automatically using ImageTS software.

### Social interaction test in home cage

The social interaction monitoring system comprised a home cage and a filtered cage top with an infrared video camera (31 × 19 × 30 cm; O'Hara & Co., Tokyo). Two mice of the same genotype that had been housed separately were placed together in the home cage. To evaluate social interaction, their location was monitored for 1 week. Output from the video camera was fed into a computer, and images from each cage were captured at a rate of 1 frame per sec. The monitoring system detects body of mice as “object(s)” in each captured image (see Miyakawa et al., 2003). Social interaction was measured by counting the number of objects detected in each image: two objects indicated that the mice were not in contact with each other, and one object indicated contact between the two mice. We also measured locomotor activity by quantifying the number of pixels that changed between each pair of successive frames. Analysis was performed automatically using ImageHA software.

### Data analysis

Behavioral data were analyzed automatically through the applications (ImageLD, ImageEP, ImageSI, ImageCSI, ImagePS, ImageTS, and ImageHC) that were developed by Tsuyoshi Miyakawa (for ImageLD and ImageEP, freely available at http://www.mouse-phenotype.org/software.html) based on ImageJ program (developed by Wayne Rasband; NIH, Bethesda, ND, USA, available at http://rsb.info.nih.gov/ij/). Statistical analysis was conducted using StatView 5.0 (SAS Institute, Cary, NC, USA). Data were analyzed using two-tailed *t*-tests and two-way repeated measures ANOVAs. Values were expressed as mean ± SEM.

## Results

### General health, motor function, and nociception in *Ox1r*^−/−^ mice

**Figure 1:**
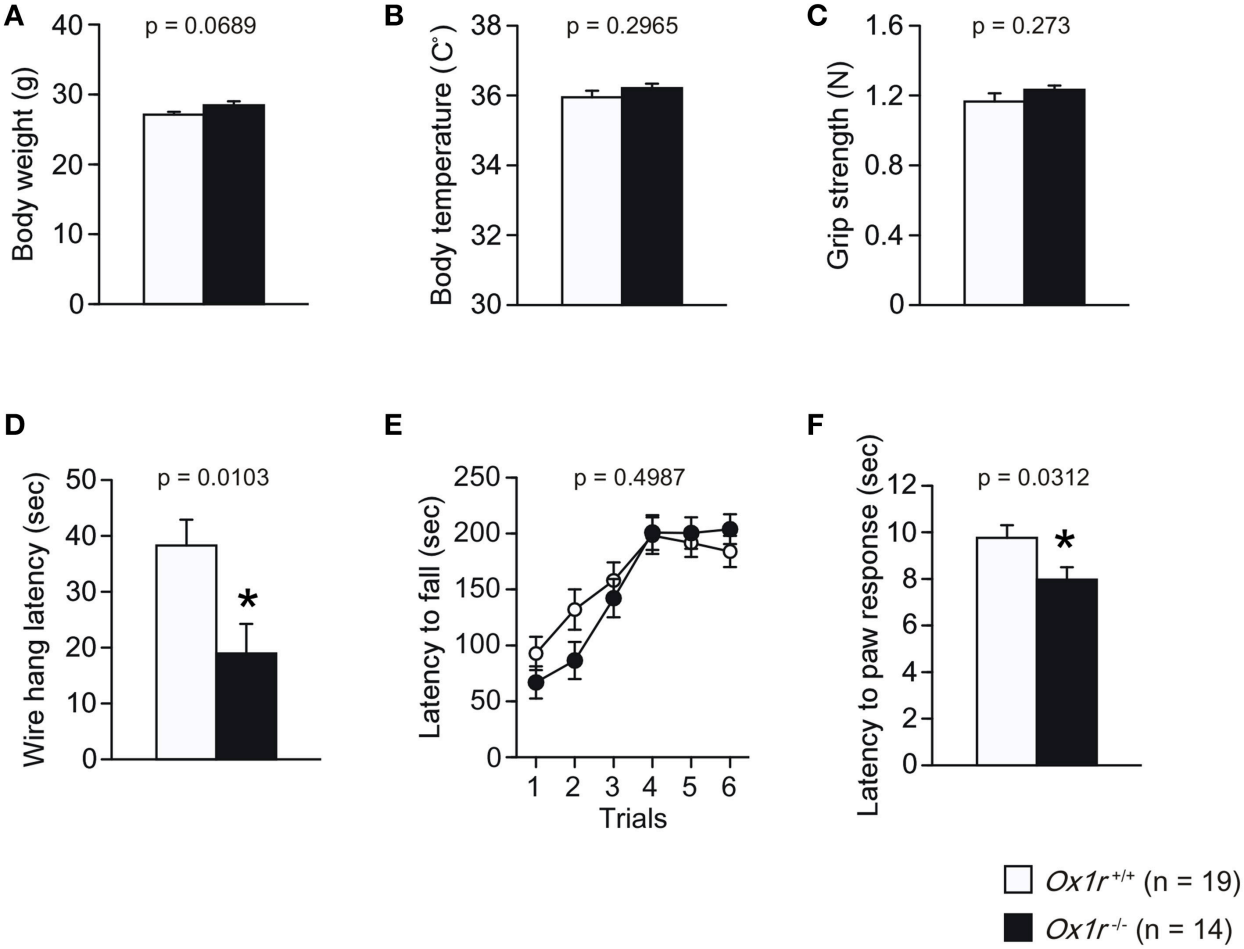
**Normal general health, neurological and motor function and nociception in ***Ox1r***^−/−^ mice**. No significant differences were found between genotypes in the body weight **(A)**, body temperature **(B)** and grip strength **(C)**. A significant reduction in the wire hang latency was observed in *Ox1r*^−/−^ mice **(D)**. No significant difference in the latency to fall off in the rotarod test was observed in *Ox1r*^−/−^ and wild-type mice **(E)**. Hot plate latency was lower in *Ox1r*^−/−^ mice **(F)**. Asterisk Indicates a significant difference from wild-type mice (*p* < 0.05).

We found no significant differences in the body weight, body temperature and grip strength between *Ox1r*^−/−^ and wild-type littermates [Figures 1A–C; for body weight, *t*_(31)_ = 1.885, *p* = 0.0689; for body temperature, *t*_(31)_ = 1.062, *p* = 0.2965; for grip strength, *t*_(31)_ = 1.116, *p* = 0.273]. There was a significant reduction of wire hang latency in *Ox1r*^−/−^ mice in the wire hang test [Figure 1D; *t*_(31)_ = 2.731, *p* = 0.0103]. There was no significant difference between *Ox1r*^−/−^ and wild-type littermates in the latency to fall off the rotating rod in the rotarod test [Figure 1E; Genotype effect, *F*_(1, 31)_ = 0.469, *p* = 0.4987; Genotype × Trial interaction, *F*_(5, 155)_ = 1.588, *p* = 0.1665]. We found significant lower latency in mutants in the hot plate test [Figure 1F; *t*_(31)_ = 2.257, *p* = 0.0312], suggesting that *Ox1r*^−/−^ mice showed hyperalgesia.

### Altered depression-like behavior and increased anxiety-like behavior in *Ox1r*^−/−^ mice

**Figure 2:**
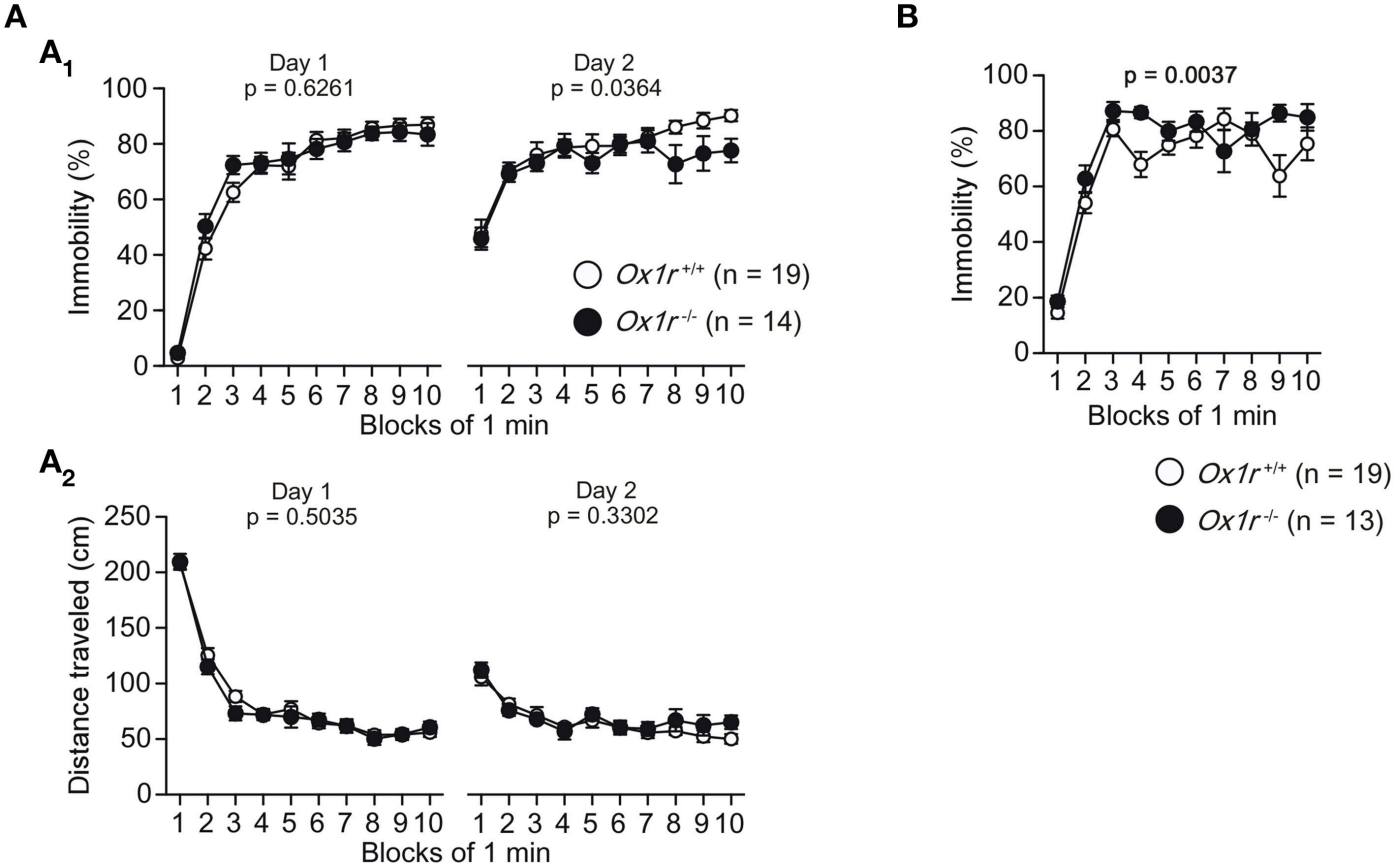
**Altered depression-like behavior in ***Ox1r***^−/−^ mice. (A)** A significant decrease in the immobility time **(A**_1_**)** on day 2 was found in *Ox1r*^−/−^ mice compared with wild-type mice, while there was no significant difference in the distance traveled **(A**_2_**)** between the genotypes in the Porsolt forced swim test. **(B)** Increased immobility time was observed in *Ox1r*^−/−^ mice compared with wild-type mice in the tail suspension test.

A significantly shorter immobility time was observed on day 2 in *Ox1r*^−/−^ mice compared with wild-type mice in the Porsolt forced swim test [Figure 2A; Genotype effect, *F*_(1, 31)_ = 4.785, *p* = 0.0364; Genotype × Time interaction, *F*_(9, 279)_ = 1.112, *p* = 0.3539], although we did not find any significant differences in the immobility time on day 1 [Genotype effect, *F*_(1, 31)_ = 0.242, *p* = 0.6261; Genotype × Time interaction, *F*_(9, 279)_ = 1.079, *p* = 0.3784] and the distance traveled on days 1 and 2 [for day 1, Genotype effect, *F*_(1, 31)_ = 0.458, *p* = 0.5035; Genotype × Time interaction, *F*_(9, 279)_ = 0.762, *p* = 0.6518; for day 2, Genotype effect, *F*_(1, 31)_ = 0.979, *p* = 0.3302; Genotype × Time interaction, *F*_(9, 279)_ = 0.713, *p* = 0.6972]. However, we found a significantly larger immobility time in *Ox1r*^−/−^ mice than wild-type mice in the tail suspension test [Figure 2B; Genotype effect, *F*_(1, 30)_ = 9.921, *p* = 0.0037; Genotype × Time interaction, *F*_(9, 270)_ = 2.116, *p* = 0.0284]. The results indicate that *Ox1r*^−/−^ mice exhibit altered behavioral responses to the forced swim and tail suspension tests, suggesting that *Ox1r* deficiency is associated with altered depression-like behavior.

**Figure 3:**
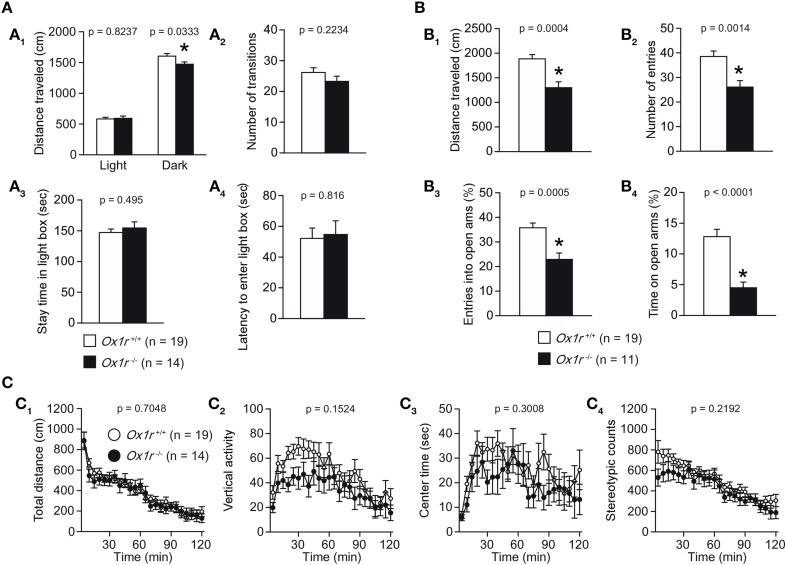
**Decreased locomotor activity and increased anxiety-like behavior in ***Ox1r***^−/−^ mice. (A)** In the light/dark transition test, no significant differences between *Ox1r*^−/−^ and wild-type mice were found in number of transitions **(A**_2_**)**, time spent in the light chamber **(A**_3_**)** and latency to enter the light chamber **(A**_4_**)**. The distance traveled in the dark chamber was significantly shorter in *Ox1r*^−/−^ mice **(A**_1_**)**. **(B)** In the elevated plus maze test, all the behavioral measures, including distance traveled **(B**_1_**)**, number of arm entries **(B**_2_**)**, percentage of entries into open arms **(B**_3_**)**, and percentage of time spent in open arms **(B**_4_**)** were significantly decreased in *Ox1r*^−/−^ mice compared with wild-type mice. **(C)** In the open field test, there were no significant differences between *Ox1r*^−/−^ and wild-type mice in the total distance traveled **(C**_1_**)**, vertical activity **(C**_2_**)**, time spent in the center **(C**_3_**)** and stereotypic counts **(C**_4_**)**. Asterisk Indicates a significant difference from wild-type mice (*p* < 0.05).

We evaluated anxiety-like behavior in *Ox1r*^−/−^ mice. In the light/dark transition test, no significant differences were observed in the distance traveled in the light chamber, number of transitions, stay time in the light chamber and latency to enter the light chamber [Figures 3A_2_–A_4_; *t*_(31)_ = 0.225, *p* = 0.8237; *t*_(31)_ = 1.242, *p* = 0.2234; *t*_(31)_ = 0.69, *p* = 0.495; *t*_(31)_ = 0.235, *p* = 0.816, respectively], although distance traveled in the dark chamber was significantly lower in the *Ox1r*^−/−^ mice as compared with wild-type mice [Figure 3A_1_; *t*_(31)_ = 2.228, *p* = 0.0333]. In the elevated plus maze test, we observed significant reductions in the distance traveled, number of arm entries, percentage of open arm entries and percentage of time spent in open arms in *Ox1r*^−/−^ mice as compared with controls [Figure 3B; *t*_(28)_ = 4.029, *p* = 0.0004; *t*_(28)_ = 3.541, *p* < 0.0014; *t*_(28)_ = 3.971, *p* = 0.0005; *t*_(28)_ = 4.814, *p* < 0.0001, respectively].

In the open field test, although there were no significant differences in distance traveled, center time, and stereotypic counts (Figures 3C_1_,C_3_,C_4_) between mutant and control mice, tendency of reduction in the number of vertical activities was observed in mutant mice (Figure 3C_2_). Decreased locomotor activity was consistently observed in other paradigms including the light/dark transition test (Figure 3A_1_), elevated plus maze test (Figure 3B_1_) (number of entries; *p* = 0.0014, entries into open arms; *p* = 0.0005, distance traveled; *p* = 0.0004, time in open arms; *p* = 0.0001).

### Increased acoustic startle response and decreased sensorimotor gating function in *Ox1r*^−/−^ mice

**Figure 4:**
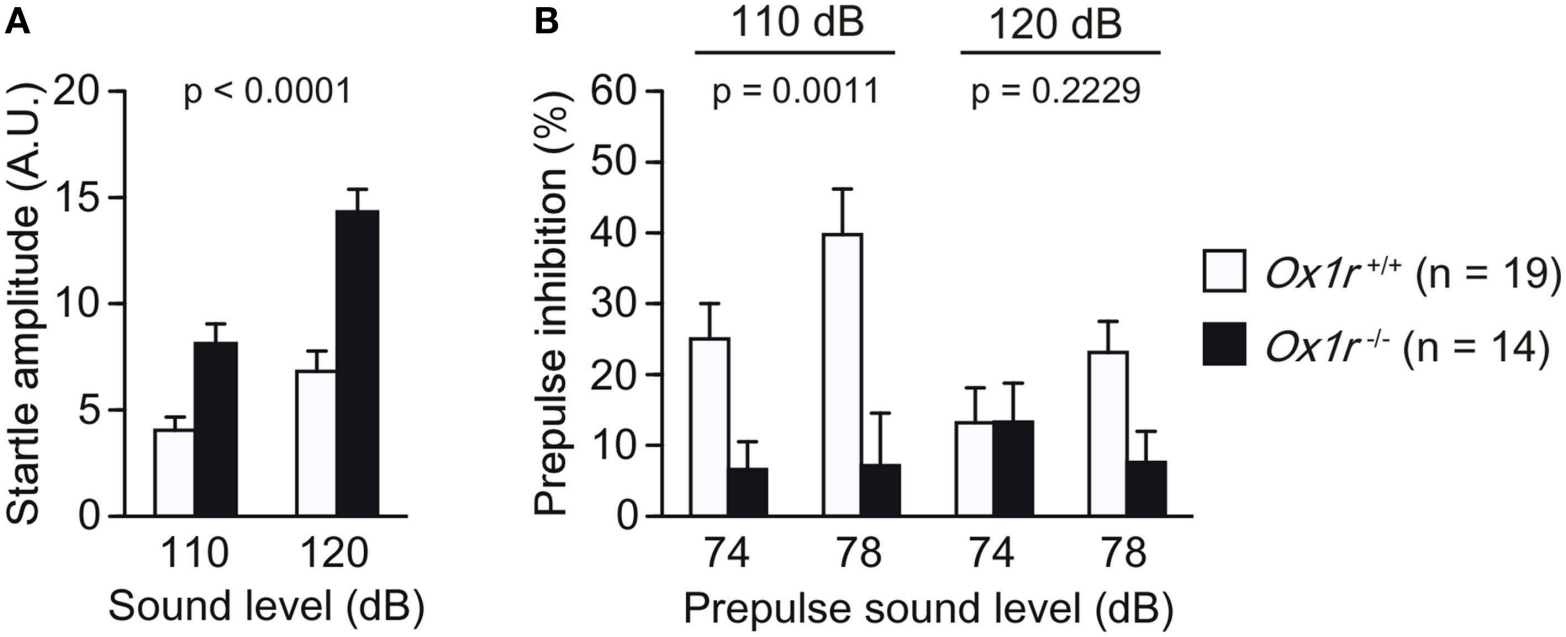
**Decreased prepulse inhibition of ***Ox1r***^−/−^ mice**. Increased startle response **(A)** and decreased prepulse inhibition **(B)** were found in mutant mice.

*Ox1r*^−/−^ mice displayed significantly greater startle response to the 110 dB and 120 dB stimuli [Figure 4A; Genotype effect, *F*_(1, 31)_ = 23.952, *p* < 0.0001; Genotype × Stimulus interaction, *F*_(1, 31)_ = 13.754, *p* = 0.0008] and lower prepulse inhibition response to 110 dB stimulus [Figure 4B; Genotype effect, *F*_(1, 31)_ = 12.906, *p* = 0.0011; Genotype × Prepulse interaction, *F*_(1, 31)_ = 2.422, *p* = 0.1298] and to 120 dB stimulus [Figure 4B; Genotype effect, *F*_(1, 31)_ = 1.547, *p* = 0.2229; Genotype × Prepulse interaction, *F*_(1, 31)_ = 6.531, *p* = 0.0157] as compared with wild-type mice. These results suggest impairment of sensorimotor gating in *Ox1r*^−/−^ mice.

### Decreased social interaction in *Ox1r*^−/−^ mice

**Figure 5:**
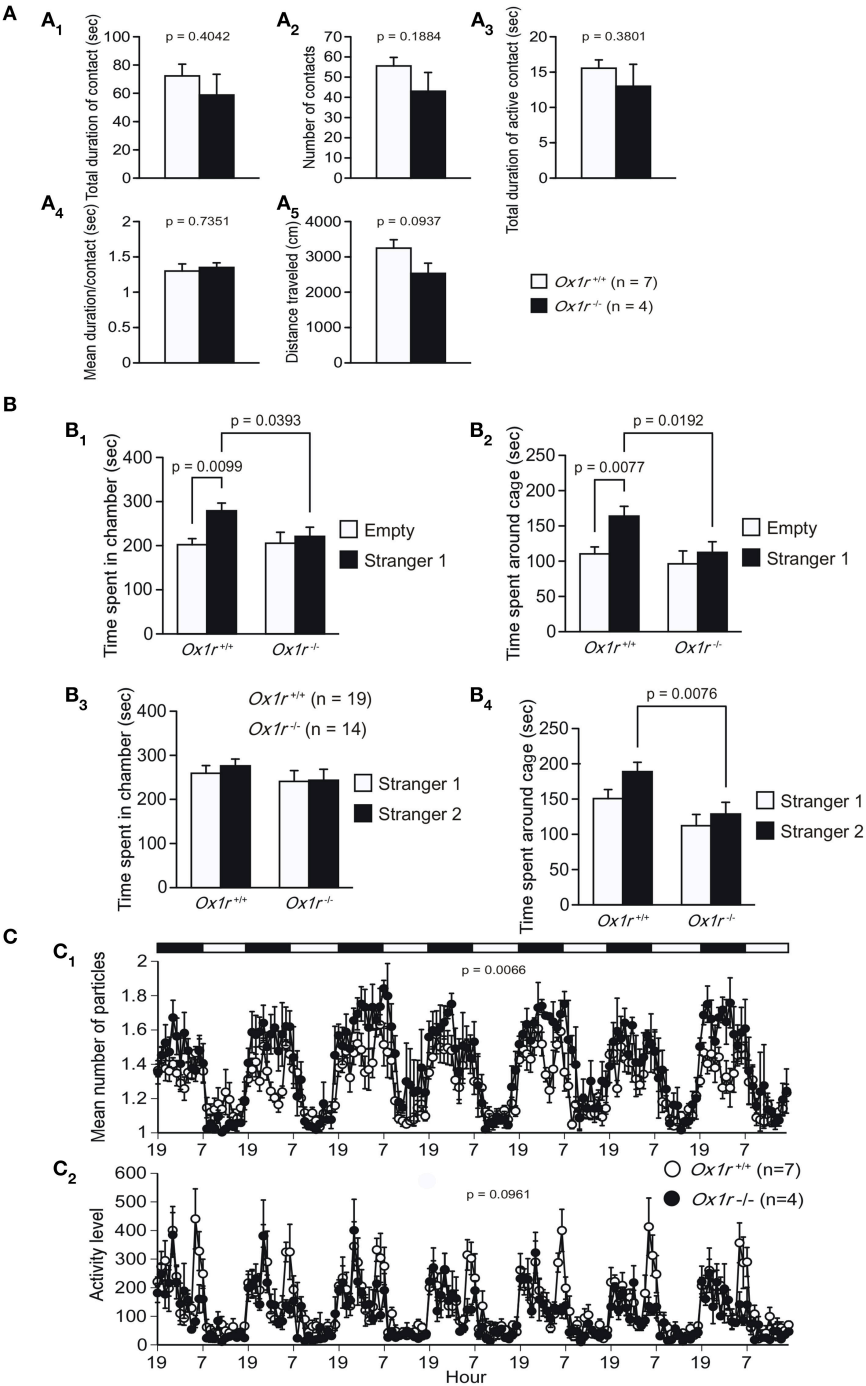
**Abnormal social behavior in ***Ox1r***^−/−^ mice**. Social interaction test in a novel environment **(A)**. Total duration of contact **(A**_1_**)**, number of contacts **(A**_2_**)**, total duration of active contact **(A**_3_**)**, and mean duration of contact **(A**_4_**)** were not significantly different between each genotype. Reduction of distance traveled was seen in mutant mice **(A**_5_**)**. In the sociability test **(B)**, control mice, but not mutant mice, spent significantly longer time in the chamber with a novel conspecific (stranger 1) than in the empty side **(B**_1_**)** and significantly longer time around the cage containing stranger 1 than that around the empty cage **(B**_2_**)**. In social novelty test, wild-type control mice tended to spend longer time around the cage containing stranger 2 than that containing stranger 1, but *Ox1r^−/−^* mice did not show this tendency **(B_3_)**. *Ox1r^−/−^* mice showed significantly shorter time spent around the cage with stranger 2 when compared with control mice **(B_4_)**. In a 24-h home cage social interaction test **(C)**, mean number of objects **(C_1_)** and activity level **(C_2_)** were lower in *Ox1r^−/−^* mice than control mice.

In the social interaction test, we observed decreased distance traveled in *Ox1r*^−/−^ mice as compared with wild-type controls although the difference between the genotypes did not reach a significance level [Figure 5A; *t*_(9)_ = 1.874, *p* = 0.0937]. The statistical analysis of the total duration of contact, number of contacts, total duration of active contact, and mean duration per contact did not show any significant differences between genotypes [Figure 5A; *t*_(9)_ = 0.875, *p* = 0.4042; *t*_(9)_ = 1.423, *p* = 0.1884; *t*_(9)_ = 0.923, *p* = 0.3801; *t*_(9)_ = 0.349, *p* = 0.7351, respectively].

In the sociability and social novelty preference test, we found several differences between *Ox1r*^−/−^ mice and wild-type control mice. In the sociability test, wild-type mice showed significantly longer time spent in a chamber containing a novel conspecific (stranger 1) in a wire cage as compared with that spent in a chamber with an empty cage [Figure 5B_1_; *t*_(18)_ = 2.882, *p* = 0.0099] and also significantly longer time spent around the wire cage containing stranger 1 than that spent around the empty cage [Figure 5B_2_; *t*_(18)_ = 3.001, *p* = 0.0077]. However, *Ox1r*^−/−^ mice showed similar contact times for both cages [for time spent in each chamber, *t*_(13)_ = 0.417, *p* = 0.6833; for time spent around each cage, *t*_(13)_ = 0.656, *p* = 0.5234]. Moreover, *Ox1r*^−/−^ mice exhibited significantly decreased social contact than wild-type mice [for time spent in chamber with stranger 1, *t*_(31)_ = 2.152, *p* = 0.0393; for time spent around cage with stranger 1, *t*_(31)_ = 2.47, *p* = 0.0192]. After this session, we introduced another novel conspecific (stranger 2) to the mice (social novelty test). In this paradigm, wild-type control mice tended to spend longer time around the cage containing stranger 2 than that containing stranger 1 [Figure 5B_3_; *t*_(18)_ = 1.765, *p* = 0.0946], although there was no difference between time spent in the chamber containing stranger 1 and time spent in the chamber containing stranger 2 in the control mice [*t*_(18)_ = 0.515, *p* = 0.6127]. In contrast, the time spent around each cage and time spent in each chamber were statistically indistinguishable between stranger 1 side and stranger 2 side [Figure 5B_4_; *t*_(13)_ = 0.633, *p* = 0.5374; *t*_(13)_ = 0.06, *p* = 0.9528, respectively]. *Ox1r*^−/−^ mice showed shorter time spent around the cage with stranger 2 when compared with control mice [for time spent in chamber with stranger 2, *t*_(31)_ = 1.174, *p* = 0.2493; for time spent around cage with stranger 2, *t*_(31)_ = 2.857, *p* = 0.0076].

In the 24-h home cage social interaction test, mean number of objects was significantly higher in *Ox1r*^−/−^ mice [Figure 5C_1_; Genotype effect, *F*_(1, 9)_ = 12.312, *p* = 0.0066; Genotype × Time interaction, *F*_(167, 1503)_ = 1.555, *p* < 0.0001]. These observations indicate that *Ox1r*^−/−^ mice showed decreased social behavior compared with wild-type mice. In addition, locomotor activity (Figure 5C_2_) was lower in *Ox1r*^−/−^ mice in the overall period [Genotype effect, *F*_(1, 9)_ = 3.453, *p* = 0.0961; Genotype × Time interaction, *F*_(167, 1503)_ = 1.337, *p* = 0.0041], light period [Genotype effect, *F*_(1, 9)_ = 10.78, *p* = 0.0095; Genotype × Time interaction, *F*_(83, 747)_ = 0.94, *p* = 0.6294], and dark period [Genotype effect, *F*_(1, 9)_ = 2.138, *p* = 0.1777; Genotype × Time interaction, *F*_(83, 747)_ = 1.663, *p* = 0.0004].

## Discussion

OX1R is expressed in the prefrontal and infralimbic cortex, hippocampus, DR, VTA, LC, and LDT/PPT, regions implicated in cognition, maintenance of sleep/wakefulness states, and regulation of emotion, the reward system and energy homeostasis (Trivedi et al., 1998; Lu et al., 2000; Marcus et al., 2001; Tsujino and Sakurai, 2009; Mieda et al., 2011).

Recently, several orexin receptor antagonists have been developed (Mieda and Sakurai, 2013). These agents are useful in pharmacological experiments aimed at identifying the physiological roles of orexins. Recent studies using these selective antagonists suggest that OX1Rs are involved in a broad range of functions including emotion, reward and autonomic regulation (Sakurai, 2014). Currently available information about functions of OX1R have been mostly obtained by these pharmacological studies using OX1R-selective antagonists, and only limited information is currently available regarding the phenotype of OX1R-deficient animals. Deletion of the OX1 receptor had no remarkable effect on sleep-wake behavior, while OX2 receptor-deficient mice showed inability to maintain wakefulness during the active phase (Sakurai, 2007; Hondo et al., 2010). As diffusion of antagonists from cerebrospinal fluid into tissues might vary among regions, pharmacological studies may not necessarily inform us about how orexin acts in physiological conditions, and because the currently available compounds are competitive antagonists, blockade of receptors by these agents is not complete. Likewise, overdosing might affect the receptor selectivity of subtype-selective antagonists. Therefore, the behavioral phenotype of these mice would be important for further understanding the physiological role of OX1R. We here conducted a comprehensive behavioral battery in *Ox1r*^−/−^ mice (Figure 1).

The general characteristics of *Ox1r*^−/−^ mice did not reveal any significant difference in their body weight, body temperature and motor function. The wire-hang test showed mild abnormality in *Ox1r*^−/−^ mice (Figure 1E). The decreased time in the wire-hang test might be related to depression-like behavior in *Ox1r*^−/−^ mice (Scott et al., 2011). These mice showed increased sensitivity to a thermal stimulus, suggesting that OX1R plays a role in control of pain sensation (Figure 1F), consistent with the previous report showing that orexin exhibits antinociceptive actions through acting on OX1R in the periaqueductal gray (Ho et al., 2011).

Present finding showed that *Ox1r*^−/−^ mice showed reduced depression-like behavior in the Porsolt forced swim test (Figure 2A), which is consistent with a part of the findings of another study (Scott et al., 2011). However, our tail suspension test exhibited *Ox1r*^−/−^ mice exhibited increased immobility as compared with wild-type (Figure 2B), rather suggesting increased depression-like behavior. These observations suggest *Ox1r*^−/−^ mice showed altered depression-like behavior depending on the paradigms and contexts, and OX1R plays complex roles in regulating the mood. OX1R is known to be expressed in regions of brain implicated in mood regulation, including the prefrontal and cingulate cortex, hippocampus, striatum, amygdala, and monoaminergic nuclei in the brain stem (Marcus et al., 2001; Mieda et al., 2011). Deficiency of OX1R in some of these regions might be related to the phenotype. A recent study showed that *Ox1r*^−/−^ mice exhibited reduced depression-like behavior (Scott et al., 2011). Some publications indicated that OX1R antagonists produced enhancement of depression-like behavior, while orexin A administration induces anti-depressive-like effects (Ito et al., 2008; Scott et al., 2011), which is consistent with our results showing that OX1R might be involved in the regulation of depression-like behavior.

Orexin neurons project to many brain regions implicated in mood (Peyron et al., 1998), and OX1R has also been found to be expressed in brain regions including the hippocampus, VTA and prefrontal cortex, which might possibly be responsible for the anti-depressant like responses (Nestler et al., 2002).

Orexin neurons innervate monoaminergic neurons such as noradrenergic neurons in the LC, dopaminergic neurons in the VTA, and histaminergic neurons in the TMN (Date et al., 1999; Nambu et al., 1999; Soya et al., 2013). Monoaminergic neuronal systems are involved in mood regulation as well as in the regulation of sleep and wakefulness. *In vitro* studies suggested that noradrenergic neurons of the LC (Horvath et al., 1999), serotonergic neurons of the DR (Brown et al., 2002; Ishida et al., 2002; Liu et al., 2002), and histaminergic neurons of the TMN (Bayer et al., 2001; Eriksson et al., 2001; Yamanaka et al., 2002) are all potently activated by orexins. OX1R is exclusively expressed in noradrenergic LC neurons, while OX2R is expressed in histaminergic neurons in the TMN (Mieda et al., 2011). Both receptors are expressed in DR serotonergic neurons. These findings indicate that orexins regulate the monoaminergic systems, which are important regulators of mood and emotion. Deficiency of OX1R-mediated regulation in these neurons might result in dysregulation of monoaminergic neurons, leading to the depression/anxiety-like phenotype found on *Ox1r*^−/−^ mice.

Our results indicate that OX1R is involved in regulation of anxiety as well as mood. Sleep and wakefulness are regulated by the monoaminergic system, which includes noradrenergic neurons of the LC, serotonergic neurons of the DR, and histaminergic neurons of the TMN, which diffusely innervate the thalamus, brainstem and cerebral cortex of the brain (Sakurai, 2007). These neurons also modulate mood and arousal. Some studies showed that chronic OX1R antagonism may produce depression-like behavior (Adidharma et al., 2012; Castillo-Ruiz et al., 2013; Deats et al., 2014), consistent with our present data. OX1R is also found in the hippocampus, which might be another candidate site of mood regulation. Our present study suggested that intervention on this system may be a possible candidate for novel treatment of anxiety and mood disorders.

Additionally, our present study showed increased startle response to auditory stimuli and lower prepulse inhibition response in *Ox1r*^−/−^ mice. These results suggest impairment of sensorimotor gating in *Ox1r*^−/−^ mice, and suggest a possibility that OX1R-mediated signaling is involved in the pathophysiology of schizophrenia.

In the present study, we found several functions of OX1R by a comprehensive behavioral analysis of Ox1r-deficient mice. Various types of neurons in several brain regions express OX1R. In our previous study, we showed that OX1R in the LC plays a critical role in establishing emotional memory, by using a selective expression of OX1R in noradrenergic neurons in the LC (Soya et al., 2013). Similarly, functions of OX1R in particular regions will be examined by region-selective rescue and/or conditional deletion of OX1R in designated regions in near future.

## Author contributions

MA, HS, SS, MH performed experiments. TM, HS, and TS made contributions to the conception and design of the work, the acquisition and interpretation of the work, drafted the work, approved the final version to be published and agreed to be accountable for all aspects of the work.

### Conflict of interest statement

The authors declare that the research was conducted in the absence of any commercial or financial relationships that could be construed as a potential conflict of interest.
